# Sanitary Care of the Lower Animals

**Published:** 1881-01

**Authors:** 


Sanitary Care of the Lower Animals—We are glad to notice how rapidly the present popularity of sanitation and preventive medicine is extending among those who have the care of the lower animals. The erection of barns, stables, sheds and poultry houses, now often involves some other question than that of expense, or even of architectural beauty. A plea that fowls, and even swine, may have fresh air, sunlight, and exercise; that the places in which they are kept be made warm in winter and cool in summer, would once have appeared very extravagant. But it is as much economy as it is kindness to look out for the health of these animals and to put them under the best hygienic conditions. The same is true of every animal whose life has any economic importance. Under all ordinary circumstances, the surroundings which secure the most comfort to the animal, are the most economical to the owner. Even a Strasbourg goose must suffer no pain, or it will not fatten.

This appreciation of the value of sanitation, however, though increasing, is not as widely extended as it ought to be. The facts given by Professor Heath, in another part of the JOURNAL, abundantly show this. The condition of many stables in this city indicates that a very great amount of ignorance, inhumanity, and short-sightedness on the part of those who take care of live stock still exists.

